# Circular RNA hsa_circ_0049657 as a Potential Biomarker in Non-Small Cell Lung Cancer

**DOI:** 10.3390/ijms241713237

**Published:** 2023-08-26

**Authors:** Yihong Ren, Yuxin Zhao, Yanan Shan, Sixuan Li, Nan Su, Zhigang Cui, Zhihua Yin

**Affiliations:** 1 Department of Epidemiology, School of Public Health, China Medical University, Shenyang 110122, China; 2021120054@cmu.edu.cn (Y.R.); 2021120058@cmu.edu.cn (Y.Z.); ynshan@cmu.edu.cn (Y.S.); sxli@cmu.edu.cn (S.L.); 2022120051@cmu.edu.cn (N.S.); 2 School of Nursing, China Medical University, Shenyang 110122, China; zgcui@cmu.edu.cn

**Keywords:** NSCLC, NFIX, hsa_circ_0049657, biomarker

## Abstract

**Simple Summary:**

This study found that hsa_circ_0049657, a circular RNA derived from the NFIX gene, was down-regulated in NSCLC tissues and cells. In addition, we could suppress the proliferation and invasion abilities and promote apoptosis of NSCLC cells by up-regulating hsa_circ_0049657.

**Abstract:**

Non-small cell lung cancer (NSCLC) is a common lung disorder. In this study, we applied bioinformatics methods to analyze and investigate the role of the NFIX gene in NSCLC. Hsa_circ_0049657 is derived from the NFIX gene, this research aimed to verify the potential role of hsa_circ_0049657 in the development of NSCLC. The results suggested that NFIX was downregulated in most cancers. In addition, the NFIX expression in lung adenocarcinoma (LUAD) was associated with the clinicopathological stage. In LUAD, NFIX expression was associated with the degree of infiltration of most immune cells. The expression levels of hsa_circ_0049657 were significantly lower in cancerous tissues than in paracancerous tissues. Moreover, the results showed that hsa_circ_0049657 expression was downregulated in NSCLC cells. After overexpression of hsa_circ_0049657, the proliferation and migration ability of NSCLC cells were significantly inhibited and the level of apoptosis was increased. We could suppress the proliferation and invasion abilities and promote apoptosis of NSCLC cells by up-regulating hsa_circ_0049657, which might be a potential biomarker for NSCLC.

## 1. Introduction

Lung cancer is a malignancy with a high incidence and mortality rate worldwide. According to statistics estimated by the International Agency for Research on Cancer (IARC), the number of new lung cancer cases in China may have reached 3.022 million in 2020, accounting for 12.7% of the total number of malignant tumor cases and ranking first among all malignant tumors [1]. Non-small cell lung cancer (NSCLC) and small cell lung cancer (SCLC) are the two major histopathological types of lung cancer, with NSCLC accounting for approximately 80–85% of all lung cancers [2].

In recent years, immunotherapy has achieved great success in cancer treatment. For example, immune checkpoints (ICs) have been a huge success in the clinical environment [3,4,5,6]. Moreover, with the rapid development of the TCGA (The Cancer Genome Atlas program), it is more convenient to further analyze the relevance and impact of individual genes in cancer prognosis and immune infiltration. Therefore, it is necessary to identify a new diagnostic and prognostic biomarker for NSCLC.

Nuclear factor I (NFI) transcription factors regulate the expression of multiple genes involved in diverse cellular activities (e.g., proliferation, migration, and differentiation) within normal development and in disease, including cancer. NFIA, NFIB, NFIC, and NFIX are four closely related genes found on human chromosomes 1p31.2-p31.3 (NFIA), 9p24.1 (NFIB), and 19p13.3 (NFIX, NFIC). NFIA, NFIB, and NFIX play critical roles in glial and neuronal differentiation in the central nervous system [7]. In contrast, NFIC has a special involvement in dental development [8]. Only NFIX contributes to muscular growth. Changes in the expression of these genes can result in a variety of diseases, including developmental abnormalities and cancer [9]. NFIX, like other developmental transcription factors, has been found to be altered in malignancies, frequently boosting pro-tumorigenic characteristics like cell proliferation, differentiation, and migration. MAFG-AS1, for example, increases pancreatic cancer development by increasing NFIX [10]. NFIX promotes cell migration by upregulating ezrin (EZR) [11]. NFIX binds to FOXA1, controlling the expression of prostate-specific genes [12]. However, several studies have revealed that NFIX can also have a tumor suppressor role, demonstrating that NFIX has a complex and cancer-type dependent role. MiR-1290 inhibits NFIX expression by binding to it [13]. MiR-647 and miR-1914 both target NFIX and reduce its expression [14]. The role of NFIX in cancer proliferation, migration, and invasion has been linked to the expression of non-coding RNAs [15].

Circular RNAs (circRNAs), formed by a covalently closed loop, have emerged as a new hot topic in the noncoding RNA network [16]. Currently, circRNAs as a novel tumor marker have become one of the emerging research directions in the life sciences. Due to their structural properties and functional diversity, circRNAs have more potential to become tumor markers than other noncoding RNAs.

In this work, we conducted a comprehensive analysis and assessed the potential value of NFIX in cancer diagnosis and prognosis. We also performed an enrichment analysis of NFIX and studied its association with immune infiltration. Furthermore, we investigated a circular RNA, hsa_circ_0049657, derived from the NFIX gene. Li et al. reported that hsa_circ_0049657 is downregulated in acute myeloid leukemia [17]. Consequently, we organized our experiments around hsa_circ_0049657 to explore the mechanism of this circRNA in NSCLC. This research identified a novel circular non-coding RNA, hsa_circ_0049657, and validated its functions in NSCLC cells. And this study revealed a novel mechanism of NSCLC progression and suggested a potential new molecular target for NSCLC research. 

## 2. Results

### 2.1. Target Gene NFIX Expression Levels

**Figure 1:**
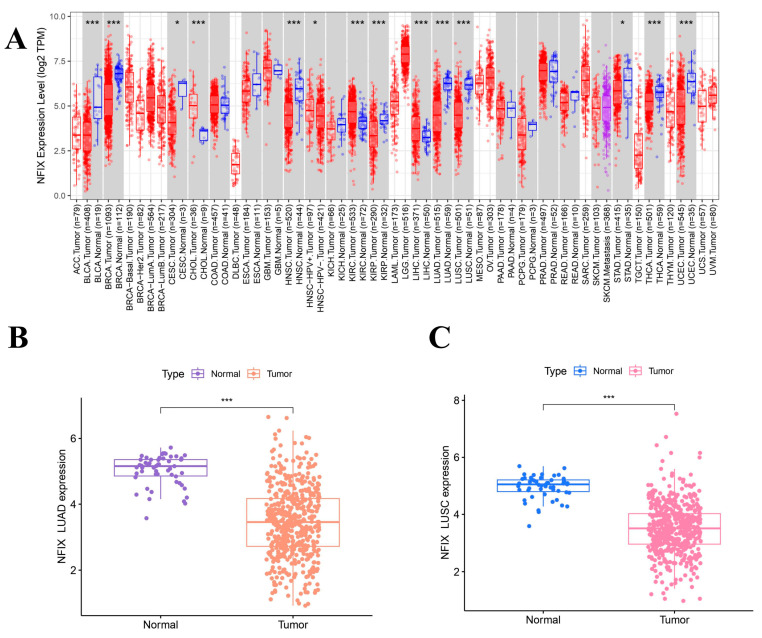

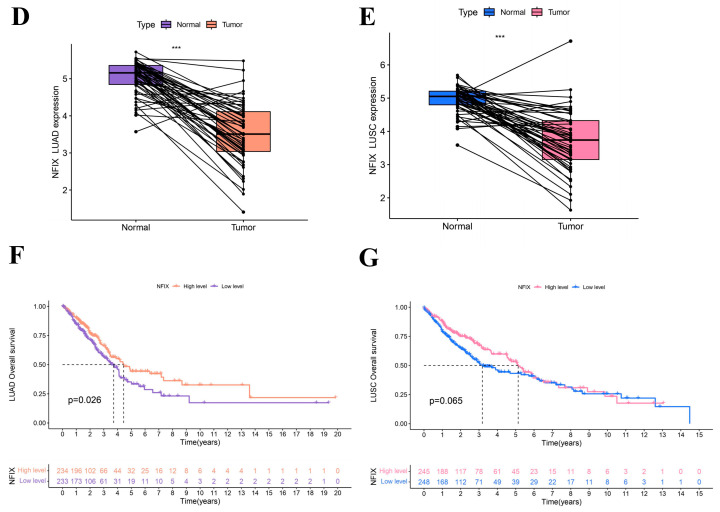
NFIX expression levels and survival analysis. (**A**) The expression of NFIX in various cancers. (**B**,**C**) Differential expression of NFIX genes in LUAD and LUSC from the TCGA database. (**D**,**E**) Paired differential expression of NFIX gene in LUAD and LUSC. (**F**,**G**) Prognostic correlation between NFIX gene expression and OS in LUAD and LUSC. * *p* < 0.05, *** *p* < 0.001.

To explore the expression of NFIX, TIMER 2.0 database analysis (Figure 1A) revealed that NFIX was downregulated in many cancers, including bladder urothelial bladder carcinoma (BLCA), breast invasive carcinoma (BRCA), cervical squamous cell carcinoma, endocervical adenocarcinoma (CESC), head and neck squamous cell carcinoma (HNSC), kidney renal papillary cell carcinoma (KIRP), LUAD, LUSC, stomach adenocarcinoma (STAD), thyroid carcinoma (THCA), and uterine corpus endometrial carcinoma (UCSC). However, NFIX was upregulated in cholangiocarcinoma (CHOL), kidney renal clear cell carcinoma (KIRC), and liver hepatocellular carcinoma (LIHC).

The results (Figure 1B,C) showed that NFIX was downregulated in tumor tissues compared to normal tissues in lung adenocarcinoma (LUAD) and lung squamous carcinoma (LUSC). The results of paired difference analyses (Figure 1D,E) in the TCGA database showed NFIX was downregulated in tumor tissues of LUAD and LUSC compared to adjacent normal tissues with *p* < 0.05.

### 2.2. Prognostic Value and Clinical Distribution of NFIX

**Figure 2:**
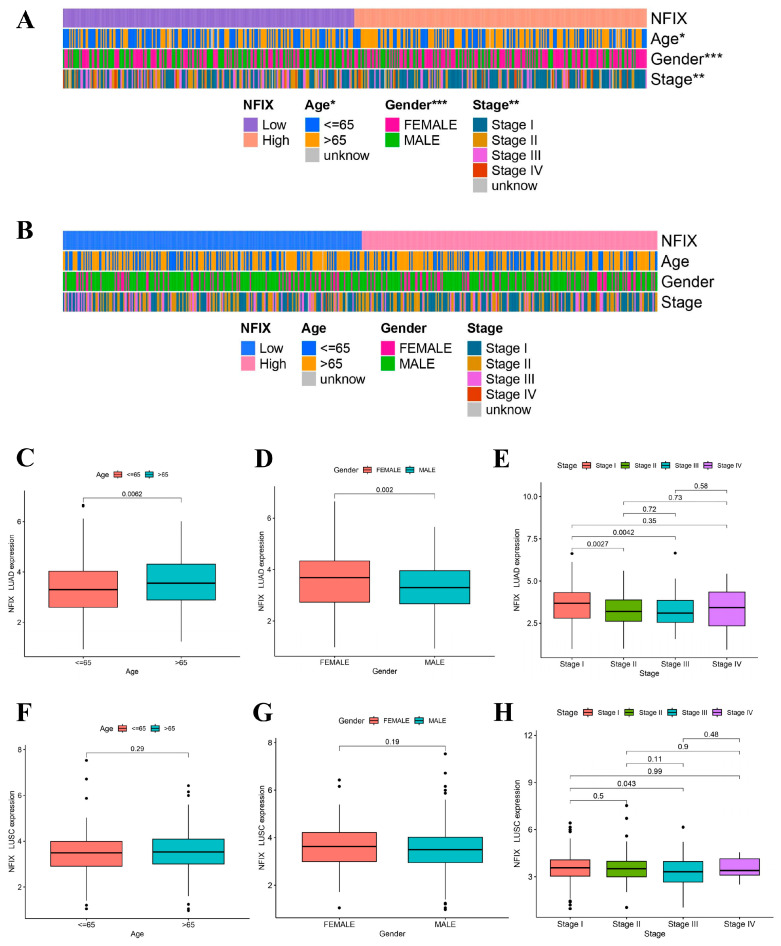
Association of NFIX expression with different clinical parameters. (**A**) Heatmap of NFIX gene expression with patient age, gender, and tumor stage in LUAD. (**B**) Heatmap of NFIX gene expression with different clinical traits in LUSC. (**C**–**E**) The link between NFIX expression and different age, gender, and tumor stage in LUAD patients. (**F**–**H**) Distribution of NFIX expression among different clinical traits in LUSC. * *p* < 0.05, ** *p* < 0.01, *** *p* < 0.001.

In this study, we found that the worse prognosis of LUAD patients with downregulated NFIX expression, suggested that NFIX may be a protective factor for LUAD prognosis (Figure 1F). However, there was no significant difference in prognosis between the high and low NFIX gene expression groups in LUSC (Figure 1G). We further investigated the relationship between NFIX expression and clinical parameters. The results indicated that NFIX gene expression had a significantly different distribution in age, gender, and tumor stage in LUAD (Figure 2A). In contrast, no significant differences were found between NFIX expression and clinical parameters in LUSC (Figure 2B). Moreover, we also visualized the distribution of NFIX expression through box plots (Figure 2C–H). The results showed that there were significant differences in NFIX expression levels between stages I and II, and stage III in LUAD, and that the higher the stage, the lower the NFIX expression (Figure 2E).

### 2.3. ESTIMATE and CIBERSORT Method

**Figure 3:**
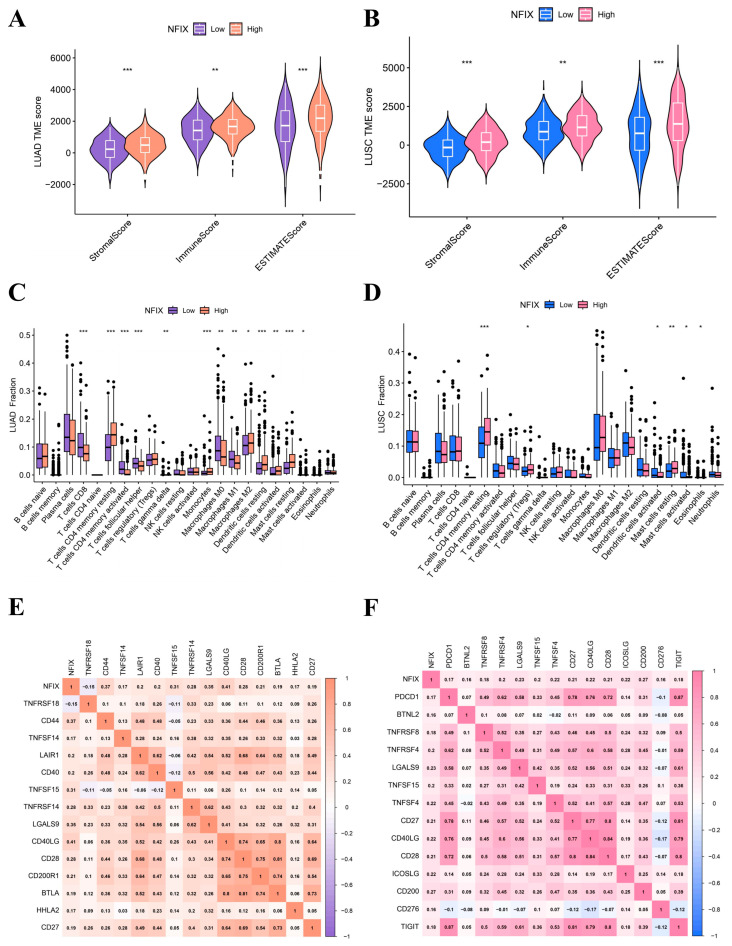
NFIX gene expression link with TME and ICs. (**A**,**B**) Differences in the three scores between high and low NFIX expression groups in LUAD and LUSC. (**C**,**D**) The percentage difference in 22 TICs between high- and low-expression groups for NFIX in LUAD and LUSC. (**E**,**F**) Correlation analysis of NFIX gene expression and ICs in LUAD and LUSC. * *p* < 0.05, ** *p* < 0.01, *** *p* < 0.001.

The ESTIMATE algorithm results revealed that NFIX expression was positively correlated with stromal score, immune score, and estimate score in LUAD (Figure 3A) and LUSC (Figure 3B). This chapter suggested that NFIX expression may be related to TME. It was now necessary to explain the relationship between NFIX and the proportion of 22 TICs using the CIBERSORT method. In LUAD, NFIX gene expression was significantly positively correlated with immune cells such as T cells CD4 memory resting, monocytes, dendritic cells activated, and dendritic cells resting and negatively correlated with immune cells such as T cells CD8, T cells CD4 memory activated, T cells follicular helper, T cells gamma delta, etc. (Figure 3C). In LUSC, NFIX gene expression was significantly positively correlated with T cells CD4 memory resting and T cells regulatory (Tregs), while it was significantly negatively correlated with dendritic cells activated, mast cells activated (Figure 3D).

### 2.4. ICs Correlation Analysis

Interestingly, NFIX was positively correlated with most of the screened immune checkpoint genes in LUAD (Figure 3E). The correlation coefficient between NFIX and CD40LG was the largest (cor = 0.41). In LUSC, NFIX was positively correlated with the screened immune checkpoint genes (Figure 3F), among which the correlation coefficient between NFIX and CD200 was the largest (cor = 0.27). Overall, these results indicated that NFIX may have potential as an immunotherapy target in LUAD and LUSC.

### 2.5. Enrichment Analysis

**Figure 4:**
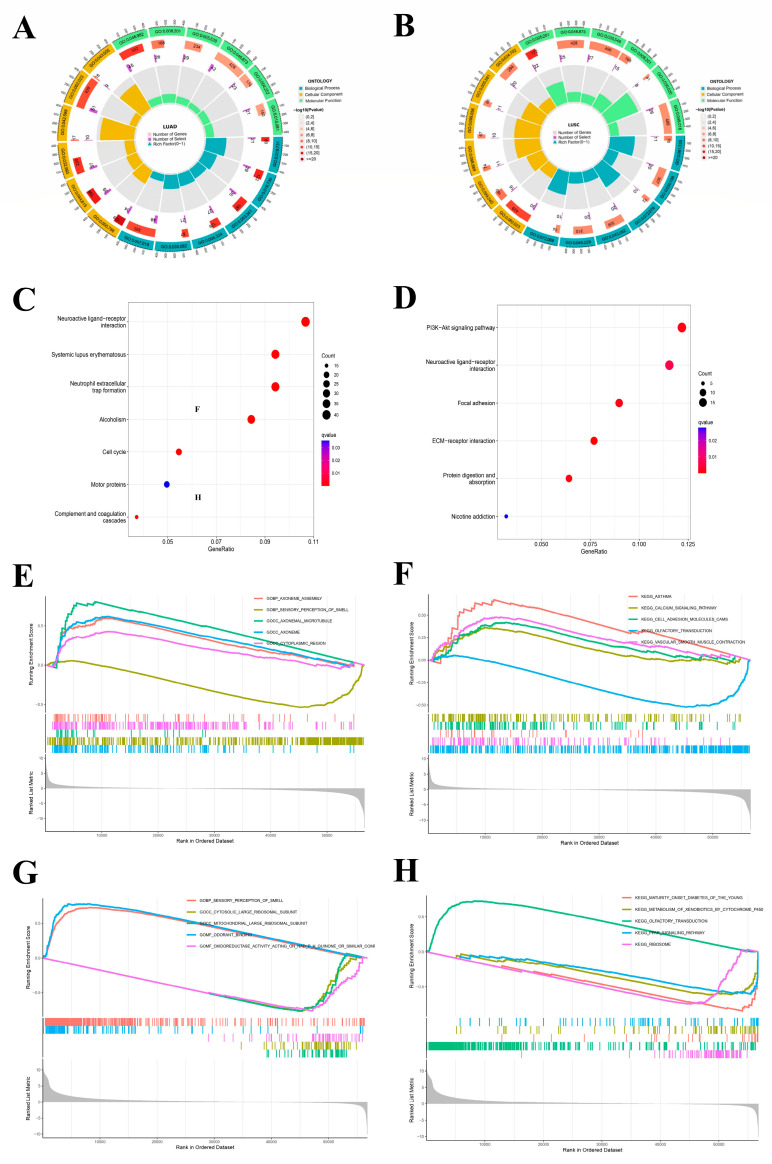
GO, KEGG and GSEA enrichment analysis of NFIX. (**A**,**B**) GO enrichment analysis of NFIX gene in LUAD and LUSC. (**C**,**D**) KEGG pathway enrichment analysis of NFIX gene in LUAD and LUSC. (**E**,**F**) GSEA analysis of the samples with different expression groups of LUAD patients. (**G**,**H**) GSEA of the samples with different expression groups of LUSC patients.

GO enrichment was meant to study NFIX from three different aspects: BP, CC, and MF. The LUAD enrichment analysis results (Figure 4A) showed that NFIX was significantly enriched in BP related to microtubule-based movement (GO: 0,007,018) and cilium movement (GO: 0,003,341). It was mainly enriched in the collagen-containing extracellular matrix (GO: 0,062,023) and protein-DNA complex (GO: 0,032,993) of CC. The MF study observed that NFIX was primarily associated with protein heterodimerization activity (GO: 0,046,982) and metal ion transmembrane transporter activity (GO: 0,046,873). The next section of the survey was concerned with KEGG enrichment. This research plotted KEGG enrichment results in the diagram (Figure 4C). The most significant pathway was the neuroactive ligand receptor interaction, among which other pathways were systemic lupus erythematosus, neutrophil extracellular trap formation, alcoholism, and cell cycle.

Then, in LUSC (Figure 4B), the enrichment results showed that NFIX was significantly enriched in BP related to an extracellular matrix organization (GO: 0,030,198), extracellular structure organization (GO: 0,043,062), and external encapsulating structure organization (GO: 0,045,229). It was mainly enriched in the collagen-containing extracellular matrix of CC (GO: 0,062,023). The MF study observed that NFIX is associated with signaling receptor activator activity (GO: 0,030,546), receptor ligand activity (GO: 0,048,018), and metal ion transmembrane transporter activity (GO: 0,046,873). The KEGG enrichment results showed that the most significant pathway was the PI3K-Akt signaling pathway and neuroactive ligand receptor interaction (Figure 4D). In the same way, we performed GSEA, and Figure 4E–H shows the top 5 significant enrichments for high- and low-expression groups in the GO and KEGG datasets, respectively.

### 2.6. CancerMine Database Correlation Analysis

**Figure 5:**
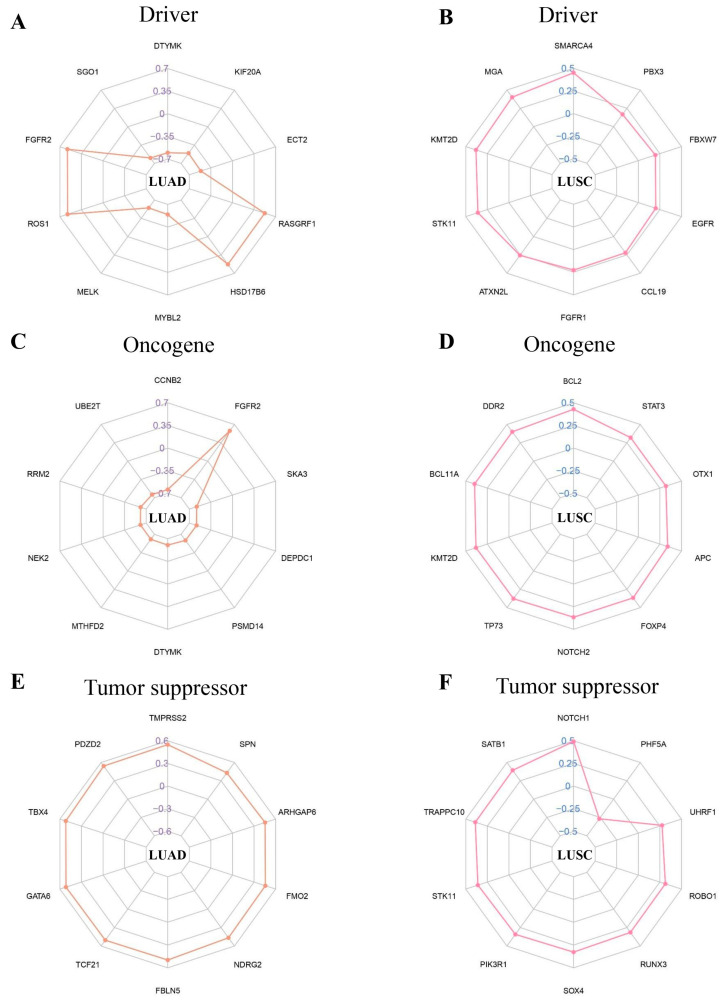
Correlation coefficient radar chart. (**A**,**B**) Correlation between driver genes and the NFIX gene. (**C**,**D**) Oncogenes associated with the NFIX gene. (**E**,**F**) Tumor suppressors associated with the NFIX gene.

In this study, we discovered that the NFIX gene has a variety of connections with different driver genes, oncogenes, and tumor suppressors in LUAD and LUSC. Our research found that in LUAD, NFIX was significantly negatively correlated with driver genes such as DTYMK and SGO1 (Figure 5A). At the same time, the expression of multiple oncogenes of NFIX was also significantly negatively correlated, except FGFR2 (Figure 5C). The analysis of tumor suppressors showed that NFIX had a positive correlation with different tumor suppressors, among which TMPRSS2 had the highest correlation coefficient (Figure 5E). In LUSC, this study found that NFIX was positively correlated with a variety of driver genes, oncogenes, and tumor suppressors, but NFIX was negatively correlated with the expression of the tumor suppressor PHF5A gene (Figure 5B,D,F).

### 2.7. Expression Levels of Hsa_circ_0049657 in NSCLC Tissues and Cells

**Figure 6:**
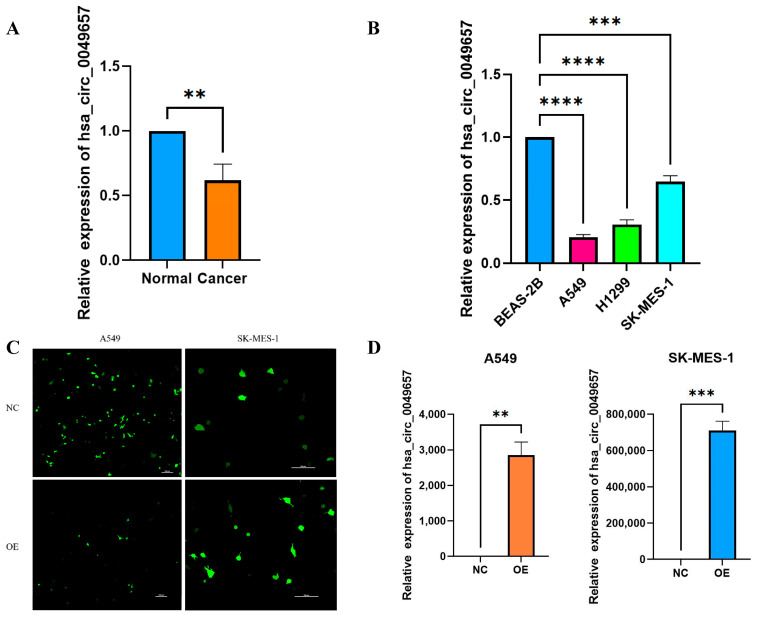

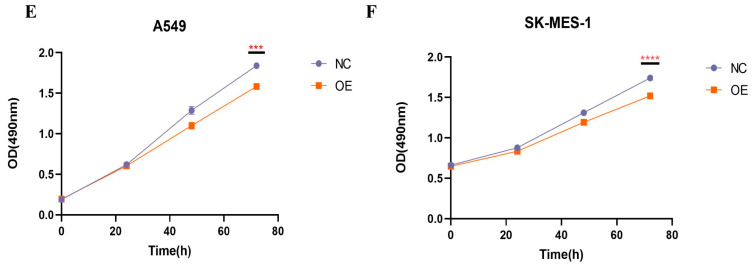
The results of qRT-PCR, transfection efficiency, and proliferation assay. (**A**) The expression of hsa_circ_0049657 in cancerous and paracancerous tissues of NSCLC patients. (**B**) The expression of hsa_circ_0049657 in NSCLC cell lines and human lung normal epithelial cells BEAS-2B. (**C**) Confocal microscopy of A549 and SK-MES-1 cells transfected with hsa_circ_0049657 overexpression plasmid and negative control. (**D**) The expression of hsa_circ_0049657 in A549 and SK-MES-1 cells transfected with overexpression plasmid. (**E**) Overexpression of hsa_circ_0049657 regulates the growth curve of the A549 cell line. (**F**) Overexpression of hsa_circ_0049657 regulates the growth curve of the SK-MES-1 cell line. NC: negative control; OE: overexpression. ** *p* < 0.01; *** *p* < 0.001; **** *p* < 0.0001.

The above bioinformatics analysis had explored the effects of NFIX in NSCLC, and it was now necessary to investigate the expression and function of the circular RNA created by NFIX (Hsa_circ_004965) in NSCLC. The qRT-PCR was first conducted to detect hsa_circ_0049657 expression in 25 NSCLC patients’ tissues. Hsa_circ_0049657 expression was significantly lower in NSCLC tissue samples than in adjacent normal tissues, with *p* < 0.01 (Figure 6A). Furthermore, cellular-level qRT-PCR results also demonstrated (Figure 6B) that hsa_circ_0049657 was significantly down-regulated in A549, H1299, and SK-MES-1 cells compared to BEAS-2B cells (*p* < 0.001).

### 2.8. Biological Functions Played by Hsa_circ_0049657 Overexpression in NSCLC Cells

**Figure 7:**
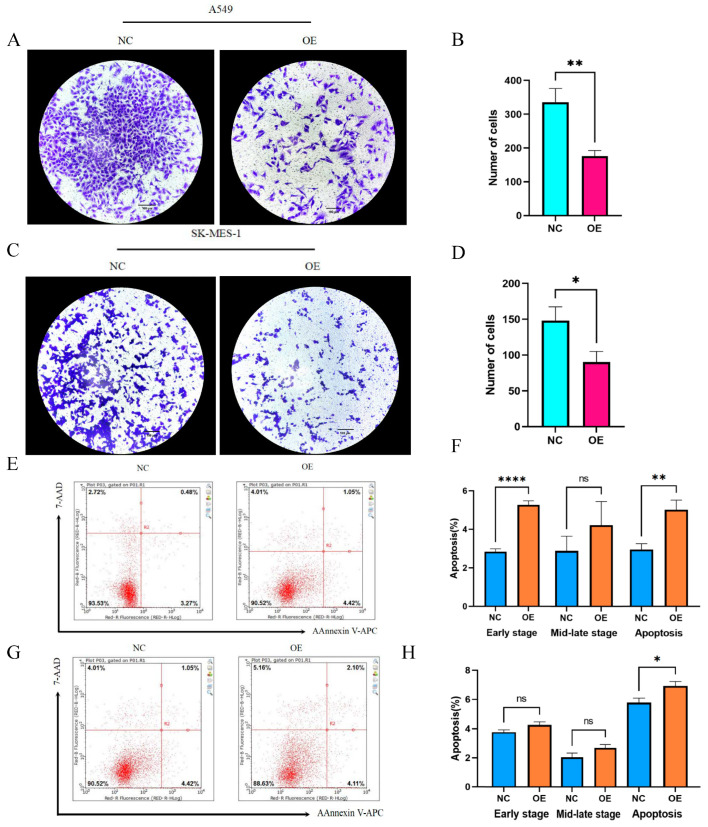
The results of the migration assay and apoptosis assay. (**A**–**D**) The transwell assay showed that the number of migrated cells was reduced via overexpression of hsa_circ_0049657 in A549 and SK-MES-1 cells. (**E**–**H**) The overexpression of hsa_circ_0049657 regulated the level of apoptosis in A549 and SK-MES-1 cells. NC: negative control; OE: overexpression. * *p* < 0.05; ** *p* < 0.01; **** *p* < 0.0001.

Having discussed the expression of hsa_circ_0049657, the final section of this paper addressed its role in NSCLC cell proliferation, invasion, and apoptosis. In our work, the A549 and SK-MES-1 cell lines were chosen for the overexpression of hsa_circ_0049657. QRT-PCR was utilized to verify the transfection efficiency (Figure 6C,D). We performed an MTS assay to explore the exact role of hsa_circ_0049657 in the proliferation of NSCLC cells. As shown in Figure 6E,F, the proliferation of A549 and SK-MES-1 cells was remarkably suppressed after overexpression of hsa_circ_0049657. Another significant aspect of hsa_circ_0049657 was cell invasion, and our transwell assay results revealed that the number of migrated A549 and SK-MES-1 cells was remarkably reduced after overexpression of hsa_circ_0049657 (Figure 7A–D). Finally, we investigated the influence of hsa_circ_0049657 on apoptosis in NSCLC cells. The apoptosis levels of A549 and SK-MES-1 cells were significantly increased after overexpression of hsa_circ_0049657 (Figure 7E–H). In addition, the results (Figure 7E,F) suggested that overexpression of hsa_circ_0049657 may have some promotional effects on the early stage of apoptosis in A549 cells.

## 3. Discussion

Our study found that NFIX was significantly downregulated in both LUAD and LUSC while correlating with LUAD staging. Moreover, prognostic analysis indicated that decreased NFIX was associated with worse OS in LUAD. Based on the above, we speculated that NFIX may act as a biomarker in the development and progression of LUAD.

The tumor microenvironment (TME) is defined as the complex and rich multicellular environment in which a tumor develops. The TME typically comprises immune cells, including T and B lymphocytes; stromal cells, such as cancer-associated fibroblasts (CAF); the extracellular matrix (ECM) and other secreted molecules; and the blood and lymphatic vascular networks, which are collectively enmeshed and in communication with each other and with the heterogeneous cancer cells themselves [18]. The TME plays an important role in cancer recurrence and drug resistance [19,20,21,22,23]. The immune cells of the TME are correlated with the effect of immunotherapy, including CD4 T cells, CD8 T cells, etc. [24,25,26,27]. Based on the results of this analysis, we hypothesize that NFIX is associated with the level of immune cell infiltration and may serve as a potential immunotherapy-related biomarker in NSCLC.

As a member of the NFI family, NFIX could act as a transcriptional switch from embryonic to fetal myogenesis [28] and could regulate both proliferation and migration during the development of the SVZ neurogenic niche [29]. The nuclear factor I/X (NFIX) plays an important role in cell differentiation, but its function in NSCLC is still unclear. In cases of endometrial carcinoma [30] and colorectal cancer [14], NFIX downregulation has been shown to promote proliferation, in line with our findings in NSCLC. However, overexpression of NFIX can promote proliferation and apoptosis in pancreatic cancer cells [10]. In summary, NFIX might perform various functions in different cancers.

Increasing evidence suggests that circRNAs are crucial regulators in the carcinogenesis of NSCLC. For instance, the sponging effect of circ_CDR1as on miR-7 has also been shown to affect the progression of lung cancer through modulation of NF-κB signaling [31]. Circular RNA 100146 functions as an oncogene through direct binding to miR-361-3p and miR-615-5p in NSCLC [32]. CircKEAP1 suppresses the progression of lung adenocarcinoma via the miR-141-3p/KEAP1/NRF2 axis [33].

Hsa_circ_0049657,which originated from NFIX, was significantly downregulated in NSCLC in our work. In NSCLC tissues and cell lines, hsa_circ_0049657 was significantly downregulated. Meanwhile, our in vitro experiments suggested that the overexpression of hsa_circ_0049657 may significantly suppress the proliferation and invasion abilities and promote apoptosis of NSCLC cells. However, the regulatory mechanism of hsa_circ_0049657 in NSCLC cells requires further investigation.

## 4. Materials and Methods

### 4.1. Data Collection

From the TCGA data Portal (https://portal.gdc.cancer.gov/ (accessed on 11 February 2023)), we selected LUAD (TCGA-LUAD) and LUSC samples (TCGA-LUSC) and downloaded the transcriptome data (FPKM), and clinical data. A total of 557 LUAD samples were obtained, including 54 cancer-adjacent normal samples and 503 cancer samples. Additionally, a total of 553 LUSC samples were obtained, including 51 cancer-adjacent normal samples and 502 cancer samples.

### 4.2. Differential Analysis

Our research explored the expression levels of NFIX in different tumors in the TIMER 2.0 database (http://timer.comp-genomics.org/timer/ (accessed on 15 May 2023)). This study explored the expression of NFIX in tumor tissue and normal tissue of LUAD and LUSC patients using the “limma” package in R language (version 4.2.0).

### 4.3. Prognostic and Clinical Correlation Analysis

The potential value of NFIX in cancer prognosis was analyzed via OS (overall survival) with the data from the TCGA database. The inclusion criteria for the 467 LUAD samples and 493 LUSC samples obtained from the TCGA encompassed the presence of comprehensive NFIX expression data, survival status information, and a minimum survival time exceeding zero. This study used R to combine NFIX log_2_(FPKM + 1) expression data with survival status and time. According to the Kaplan Meier method, we grouped the subjects by the median of NFIX expression to determine whether there was a difference in survival between high- and low-expression groups (*p* < 0.05). Moving on now to consider the expression levels of NFIX in different clinical trait groups and explore the relationship between NFIX expression and clinical traits in the TCGA database.

### 4.4. Tumor Microenvironment (TME) and Tumor-Infiltrating Immune Cells (TICs) Research

The section below describes the ESTIMATE and CIBERSORT methods for exploring the TME in NSCLC patients. The ESTIMATE score is obtained by adding the stromal and immune scores, which may predict tumor purity. To explore the relationship between tumor purity and NFIX expression, we compared the scores of high- and low-expression groups. In the meantime, we applied the CIBERSORT method to estimate the proportion of 22 TICs in NSCLC tumor samples. This analysis used the Wilcox ranking sum test to examine differences in the proportion of TICs between high- and low-expression groups.

### 4.5. Immune Checkpoint Correlation Analysis

In the next section, we analyzed the association between the target gene NFIX and the expression of ICs (*p* < 0.001) and visualized the results through R language.

### 4.6. Enrichment Analysis

The NFIX gene was analyzed by gene ontology (GO), the Kyoto encyclopedia of genes and genomes (KEGG), and gene set enrichment analysis (GSEA). The GO analysis mainly studies biological processes (BP), molecular functions (MF), and cellular components (CC). We enriched the NFIX in the above three aspects (*p* < 0.05 and q < 0.05), and also the enrichment results were pictured in a chart graph by the “clusterProfiler” package. And we performed KEGG analysis (*p* < 0.05 and q < 0.05) to review the link between NFIX and biological pathways. By GSEA, we could speculate which signaling pathways NFIX plays a role in. Our study divided TCGA tumor samples into high- and low-expression groups in line with the NFIX expression median. We preserved each group’s top five results with the maximum normalized enrichment score.

### 4.7. CancerMine Database Correlation Analysis

This study was based on CancerMine database mining with LUAD and LUSC on driver genes, oncogenes, and tumor suppressors. After that, we evaluated the association of different genes with the NFIX gene by calculating correlation coefficients using the R language. Finally, the top 10 most significant genes in each group were visualized in radar charts.

### 4.8. Clinical Samples

The study collected 25 NSCLC patients’ lung cancer tissue samples and adjacent standard tissue samples, including 17 cases of LUAD and 8 cases of LUSC. They consisted of 10 females and 15 males, with an age distribution of 50 to 81 years. All patients had signed the relevant informed consent. The medical ethics issues involved in the research program were reviewed and approved by the medical ethics committee.

### 4.9. Cell Culture

Human NSCLC cell lines (A549, SK-MES-1, H1299) and the regular human lung epithelial cell line (BEAS-2B) were offered by Shanghai Genechem Co., Ltd., (Shanghai, China). All cells were cytogenetically tested and authenticated before freezing. All cells culture conditions were created and maintained following Shanghai Genechem Co., Ltd.’s instructions. The F12K complete medium was used to culture A549 cells in the following ratios: 10% fetal bovine serum, 80 U/mL penicillin, 0.08 mg/mL streptomycin; MEM complete medium containing 10% fetal bovine serum was used to culture SK-MES-1 cells; BEAS-2B was cultured in DMEM complete medium containing 10% fetal bovine serum. Meanwhile, the cells were maintained in an incubator with 5% CO_2_ at 37 °C.

### 4.10. Cell Transfection

Hsa_circ_0049657 overexpression plasmid and negative control (NC) were designed and synthesized by Beijing Syngenbio Co., Ltd., (Beijing, China). Moreover, the pLV-hef1a-mNeongreen-P2A-Puro-WPRE-CMV-5’ vector was used for cloning the hsa_circ_0049657 sequence and negative control, which was transfected into A549 and SK-MES-1 cells. After 24 h of transfection, the cells were observed using a confocal microscope (NiKon, Japan) (the plasmid was designed to incorporate fluorescence, and the cells will fluoresce green when plasmids enter the cells).

### 4.11. Quantitative Real Time-Polymerase Chain Reaction (qRT-PCR)

Total RNA was extracted from NSCLC cells and tissues using the Trizol method. RNA concentration was measured using an ultraviolet spectrophotometer (IMPLEN, Germany). Subsequently, extracted RNA was reverse transcribed into cDNA according to the instructions of the PrimeScript^TM^ RT MasterMix kit (Takara, Dalian, China). PCRs were performed by using the SYBR Green method in an ABI 7500 FAST sequence detection system (Applied Biosystems). The thermal cycle was as follows: 30 s at 95 °C, 3 s at 95 °C, and 30 s at 60 °C for 40 cycles. The primer sequences used in the present study are given in Table 1. The primers were synthesized by Sangon Biotech (Shanghai) Co., Ltd., (Shanghai, China).

### 4.12. Cell Proliferation Assay

We performed an MTS assay to explore the exact role of hsa_circ_0049657 in the proliferation of NSCLC cells. To analyze the influence of hsa_circ_0049657 on the proliferation of NSCLC cells, the proliferation of transfected A549 and SK-MES-1 cells was monitored using CellTiter 96 AQueous One Solution Cell Proliferation Assay (Promega, Beijing, China). Briefly, 20 μL CellTiter 96 AQueous One Solution Cell Proliferation Assay was added to each well at 0, 24, 48, and 72 h after transfection, respectively, followed by incubation for 1 h. To finish, we measured the OD 490 using the Multifunctional Enzyme Labeler (BioTech, Hangzhou, China).

### 4.13. Cell Migration Assay

Transwell chambers with 8 μm pores were provided by Corning (Corning, NY, USA). Cells were first seeded into the upper section of a 24-well plate. Meanwhile, a complete culture solution was added to the lower chamber of culture inserts. After incubation for 24 h, these inserts were fixed with methanol for 30 min and stained with crystal violet for 8 min. Finally, invading cells were observed with an ortho-inverted microscope (Echo, San Diego, CA, USA), and then the number of cells was calculated using Image J software (version 1.8.0, National Institute of Health, Bethesda, MD, USA).

### 4.14. Cell Apoptosis Assay

The apoptosis of transfected NSCLC cells was analyzed by using the Annexin V-APC/7-AAD double-stained apoptosis detection kit (KeyGEN, Nanjing, China). After incubation for 15 min at room temperature and protection from light, the level of apoptosis was detected by flow cytometry (Millipore, MA, USA).

### 4.15. Statistical Analysis

Statistical Product and Service Solutions (SPSS) 21.0 (SPSS, Chicago, IL, USA) and PRISM 9 (GraphPad Software Inc., San Diego, CA, USA) were used for all statistical analyses. The Student’s *t*-test was used to compare the differences between two groups. *P* < 0.05 was considered statistically significant.

## 5. Conclusions

In conclusion, we discovered that NFIX exerts an essential function in the occurrence and development of NSCLC. Meanwhile, hsa_circ_0049657 (from the NFIX gene) overexpression could inhibit the proliferation and invasive ability of NSCLC cells and promote their apoptosis. We hypothesized that NFIX and hsa_circ_0049657 may be potential biomarkers for NSCLC.

## Figures and Tables

**Table 1:** Primers sequences.

Gene	Primer	Sequence (5′ to 3′)
hsa_circ_0049657	Forward	CTCGCCCAAGTCGGAATATAC
	Reverse	CTGGTAGCTGGAGTAGATCGT
GAPDH	Forward	CAGGAGGCATTGCTGATGAT
	Reverse	GAAGGCTGGGGCTCATTT

## Data Availability

The clinical datasets of patients with NSCLC in a hospital in Shenyang that were analyzed or generated during the study are available from the corresponding author upon reasonable request. The RNA-seq data used in this study can be obtained in the GDC databases of TCGA.
